# The effect of triclosan coated sutures on rate of Surgical Site Infection after hip and knee replacement: a protocol for a double-blind randomised controlled trial

**DOI:** 10.1186/1471-2474-15-237

**Published:** 2014-07-14

**Authors:** Andrew P Sprowson, Cyrus D Jensen, Nick Parsons, Paul Partington, Kevin Emmerson, Ian Carluke, Seif Asaad, Roland Pratt, Scott Muller, Mike R Reed

**Affiliations:** 1 Warwick Medical School, The University of Warwick, Coventry CV4 7AL, UK; 2 Northumbria NHS Foundation Trust, Rake Lane, North Shields, Tyne and Wear, UK

## Abstract

**Background:**

187,000 hip and knee joint replacements are performed every year in the National Health Service (NHS). One of the commonest complications is surgical site infection (SSI), and this represents a significant burden in terms of patient morbidity, mortality and cost to health services around the world. The aim of this randomised controlled trial (RCT) is to determine if the addition of triclosan coated sutures to a standard regimen can reduce the rate of SSI after total knee replacement (TKR) and total hip replacement (THR).

**Methods:**

2400 patients due to undergo a total hip or knee replacement are being recruited into this two-centre RCT. Participants are recruited before surgery and randomised to either standard care or intervention group. Participants, outcome assessors and statistician are blind to treatment allocation throughout the study. The intervention consists of triclosan coated sutures vs. standard non-coated sutures. The primary outcome is the Health protection Agency (HPA) defined superficial surgical site infection at 30 days. Secondary outcomes include HPA defined deep surgical site infection at 12 months, length of hospital stay, critical care stay, and payer costs.

**Discussion:**

To date there are no orthopaedic randomised controlled trials on this scale assessing the effectiveness of a surgical intervention, particularly those that can be translated across the surgical specialities. The results from this trial will inform evidence-based recommendations for suture selection in the management of patients undergoing total hip or knee replacement. If triclosan coated sutures are found to be an effective intervention, implementation into clinical practice could improve long-term outcomes for patients undergoing hip and knee replacement.

**Trial registration:**

Current Controlled Trials ISRCTN 17807356.

## Background

Total hip replacements (THR) and total knee replacements (TKR) are performed for relief of debilitating pain secondary to arthritis, and are the commonest forms of joint replacement performed in the UK. Over 187,000 hip and knee replacements were performed in 2012 [1] in England and Wales. Over the past decade there has been a stark rise in the number performed due to; earlier diagnosis, improved availability to healthcare resources, awareness amongst medical practitioners and patients themselves. By 2030, the demand for primary total hip replacement is estimated to grow by 174% and the demand for primary total knee replacement is projected to grow by 673% [2] in the United States.

TKR and THR are safe, with relatively low risk of complications. However, Surgical site infection (SSI) is potentially a very serious complication, which currently occurs in around 1 out of every 100 joint replacements performed [3]. A multitude of risk factors influence the development of SSIs and awareness of these will help to promote effective preventive strategies [4,5]. A surgical site is the incision made in skin by a surgeon to carry out a surgical procedure, and an infection occurs when microorganisms get into the part of the body that has been operated upon. SSI’s are defined as affecting the superficial or deeper tissues handled during the procedure [6]. This complication represents an enormous personal cost to the patient and a very significant cost to the NHS - in the region of £61 million each year [7].

The aim of closure of a surgical wound is to promote rapid healing by opposition of skin edges to leave a cosmetically acceptable scar [8]. The technique should be watertight, tension free, and should be without inverting the skin edges. Delayed wound healing especially in high risk groups, such as elderly, diabetic, immunocompromised patients can delay recovery and progress to deep wound infection. This increases morbidity, mortality, and has huge cost implications.

A commonly used absorbable braided synthetic fibre suture (Vicryl) is used ubiquitously across surgical specialities, coated with equal parts of a copolymer of lactide and glactide. This mixture forms an absorbable, adherent, non-flaking lubricant. As a plain suture, it exhibits tissue drag and “chatter” as it is tied. The suture elicits only a mild tissue reaction and was one of the first synthetic absorbable sutures developed for applications for which chromic gut could not be used. It is absorbed predictably as it retains 65% of its strength at 14 days after implantation, 40% at 21 days and absorption is complete by 70 days [9].

To enhance the antimicrobial characteristic of a suture, triclosan has been added (Vicryl plus), which although not an antibiotic, acts as a broad-spectrum antibacterial agent. Triclosan has been effectively used in consumer products for more than 30 years [10]. In vitro studies demonstrate that triclosan coated sutures create an “active zone” around the suture, inhibiting staphylococcus aureus, staphylococcus epidermidis and methicillin resistant strains of staphylococcus (MRSA and MRSE), the leading surgical site bacteria, from colonizing on the suture for a minimum of 48 hours [11-14].

The aim of this trial is to determine if using a triclosan coated suture can significantly reduce the rate of superficial SSI at 30 days after primary TKR and THR.

## Methods

The Northumbria Arthroplasty Suture Study (NASS) is an on-going two-centre double blind randomised controlled trial conducted at Northumbria NHS Foundation Trust, one of the largest elective orthopaedic centres in the UK. The study has been approved by Newcastle and North Tyneside Research Ethics Committee (07/H0901/62) and all participants provide informed http://www.controlled-trials.com/ISRCTN25633145). Johnson & Johnson (UK) has provided partial funding for this trial.

### Study duration

Recruitment into the trial began in December 2008, all participants are now recruited and the 12-month follow-up stage will be completed in early 2014.

### Study participants

Participants deemed suitable for a total hip or total knee replacement. Patients must satisfy general requirements for a THR or TKR.

### Inclusion criteria

There were no restrictions based on age, sex, previous infections and all patient requiring a total hip or total knee replacement at 1 of the 2 sites were included. All patients were screened for MRSA and had to have a negative swab prior to surgery.

### Exclusion criteria

Require revision knee or hip replacement surgery.

Unicondylar or patellofemoral knee replacement.

Are unlikely to be able to perform required clinical assessment task.

### Participant recruitment

Patients were identified by participating surgeons and approached in outpatients and at pre-assessment clinics by research associates using good clinical practice principles. Patients were provided with a patient information leaflet and a formal Invitation letter. Eligible patients then had a minimum period of a week to consider entry into the trial. Patients were recruited or given further information about the study at their pre-assessment visit. A further opportunity to recruit patients is available on admission for surgery.

All patients will be given sufficient time to accept or decline involvement. It will be made clear verbally and in written form, that they will be free to withdraw from the study at any time without affecting their routine perioperative care.

### Participant allocation

Treatment group allocation is based upon an opaque envelope randomised design utilising the date surgery is performed. This allocation is based on random monthly hospital assignment into one of the two groups, each centre providing one treatment for the whole calendar month. Envelopes were only opened at the start of a month, so allocation was not known at the time of listing. Each month the hospitals would be randomised to either control or intervention. A more optimal conventional randomisation methodology, that is randomly allocating individual study participants to one of the two treatment groups at recruitment, is not possible for this study. It was decided by the study development team that it would not be feasible or practical to attempt to do this in the selected setting. Multiple sutures with a variety of surgeon specific needle types would have been used, so a considerable number of blinded sutures would be needed, with the majority discarded. Equally we would have to store large numbers of each suture in the theatre if randomised individually. Lack of specific local support for randomisation and concerns about the impact on the credibility and fidelity of the study interventions were identified as problematic issues if individual participant randomisation were adopted. Within this study setting the surgeon was not blinded to the sutures. The study participants, research associates involved in recruitment and assessment, and clinical staff involved in the care of study participants were all blind to treatment allocation throughout the study. The statistician is also blinded.

### Interventions

#### Hip and knee replacement participants

Before the operation, all patients followed the same patient pathway from out patient appointment to operation date. The surgeon will be aware of the suture, which will be used to close all layers of the wound, including the subcutaneous skin in a subcuticular technique if deemed appropriate by the operating surgeon. If a subcuticular skin closure technique is not deemed appropriate then metal clips will be used for the skin layer. Prophylactic parenteral antibiotics prophylaxis at the start of trial was Gentamicin (4.5 mg/kg) and this was changed to Gentamicin (3 mg/kg) and Teicoplanin (400 mg) in February 2009 [15] in line with our trust prophylaxis for primary joint replacement. On the initiation of the study the latest systematic review stated there was insufficient evidence to suggest that there was a significant difference in the efficacy of cephalosporin’s compared with that of teicoplanin or penicillin-derivatives, or that a particular generation of cephalosporin’s was more effective than another [16]. Antibiotics were given as a single dose, within 30 minutes of induction.

### Standard care group

This study is pragmatic in design and we did not stipulate method of analgesia, anaesthesia, and post-operative care, however there is a well-developed enhanced recovery programme which all surgeons follow. The surgical approach, implants used and method of closure is left entirely to individual surgeon preference. Standard sutures will be used.

### Intervention group

The intervention group will receive exactly the same regimen as the standard of care group, except they received a triclosan coated suture used for wound closure. The suture contains a triclosan coating on the surface of the suture, with a potential local antibiotic action. The suture is a commercial product in common use, from the same company to maintain consistency across control and intervention arm [4].

### Risks

No additional risks for study patients were expected, since all surgical procedures carried out within NASS represent clinically established standard methods of treatment in hip and knee replacement.

### Primary outcome measure

The primary outcome is superficial SSI infection based upon Health Protection Agency (HPA) published definitions, which originate from the Centers for Disease Control and Prevention (CDC) 1992 published definition. The HPA criteria are the nationally agreed definition used within the UK, and routinely collected by the majority of UK Trusts [17]. Superficial incisional infection is defined by the HPA, as a surgical site infection that occurs within 30 days of surgery and involves only the skin or subcutaneous tissue of the incision, and meets at least one of the criteria in Table 1. To ensure accurate collection the 30-day HPA patient self reported questionnaire was recorded in all patients collected by telephone, by a blinded research nurse.

**Table 1:** Health protection agency definition of superficial surgical site infection

**Superficial incisional infection**	
SSI that occurs within 30 days of surgery, involves only the skin or subcutaneous tissue of the incision & meets at least **one** of the following criteria:	
1.	Purulent drainage from superficial incision
2.	Culture of organisms **and** pus cells present:
	fluid/tissue from superficial incision wound swab from superficial incision
3.	At least 2 symptoms of inflammation:
	Pain, tenderness, localised swelling, redness, heat **and** either:
	1) Incision deliberately opened to manage infection
	**or**
	2) Clinicians diagnosis of superficial SSI

Note: Stitch abscesses (minimal inflammation/discharge at suture point) do not classify as SSI.

### Secondary outcome measures

Deep incisional infection is defined as a surgical site infection involving the deep tissues (i.e. fascial and muscle layers) that occurs within a year, the infection appears to be related to the surgical procedure, and meets at least one of the criteria in Table 2. These criteria are those nationally defined and validated.

**Table 2:** Health protection agency definition of deep surgical site infection

**Deep incisional infection**	
SSI involving the deep tissues (i.e. fascial & muscle layers), within 30 days of surgery (or 1 year if an implant is in place) and the infection appears to be related to the surgical procedure & meets at least **one** of the following criteria:	
1.	Purulent drainage from deep incision (not organ space)
2.	Organisms from culture **and** pus cells present in:
	Fluid/tissue from deep incision or wound swab from deep incision
3.	Deep incision dehisces or deliberately opened **and** patient has at least **1** symptom of:
	Fever or localised pain/tenderness
4.	Abscess or other evidence of infection in deep incision:
	Re-operation/histopathology/radiology
5.	Clinicians diagnosis of deep incisional SSI

Note: An infection involving both superficial and deep incisional.

### Length of hospital stay

This is calculated from participant’s admission and discharge dates and is the number of nights in hospital.

### 30 and 90-day mortality

Mortality data were obtained from the Office of National Statistics (ONS). In England, deaths must be registered within 5 days. Burials and cremations cannot be conducted without this registration documentation. These deaths are recorded by the ONS and are added to the patient’s health service record.

### Medical and surgical complications

This data is recorded from hospital records during the in-patient stay, and utilising Hospital Episode Statistics data.

### Pre operative patient factors

A number of possible potential prognostic factors are being recorded, including socio-demographic factors and medical co-morbidities. Due to the importance of these factors for SSI multi-variant logistic regression analysis, adjusting for pre operative factors will be performed.

### Specific postoperative complications

An additional number of specific complications are being recorded such as Deep Vein Thrombosis (DVT) at 60 days, Pulmonary Embolism (PE) at 60 days, stroke at 30 days, Transient Ischemic Attack (TIA) at 30 days, GI Bleed at 30 days (GIB), Urinary Retention at 30 days (UR), Urinary tract infection (UTI) at 30 days, Myocardial Infarction (MI) at 30 days, Pneumonia at 30 days (RTI), and readmission rate. The blinded research nurse will identify these complications for each patient in the trial and HES data will be used as a secondary recording method.

### Sample size calculation

The primary outcome for this trial is SSI based on the HPA defined criteria at 30 days post-operation. Two thousand four hundred patients listed for primary hip or total knee replacement are being recruited into the study, and quasi randomised to the intervention arm or the standard care arm. At the initiation of the study the trusts 12 month audited rate of superficial SSI was 2.25% for hip and total knee replacement, which is within that documented in the literature. This sample size will provide 80% power to detect a reduction of SSI infection from 2.25% to 1%, at the 5% level. This difference represents a significant reduction, which would have an important clinical impact.

### Statistical analysis

A CONSORT diagram [18] will summarise participant flow through the study, documenting eligibility and recruitment, receipt of intervention or standard care as allocated, and collection of data [19]. Standard statistical summaries (e.g. medians and ranges or means and variances, dependent on the distribution of the outcome) and graphical plots showing correlations will be presented for the primary outcome measure and all secondary outcome measures. Baseline data will be summarised to check comparability between treatment arms, and to highlight any characteristic differences between those individuals in the study, those ineligible, and those eligible but withholding consent. Although missing data is not expected to be a major issue for this study, the nature and pattern of the missingness will be carefully considered — including in particular whether data can be treated as missing completely at random (MCAR). If judged appropriate, missing data will be imputed, and resulting imputed datasets will be analysed and reported, together with appropriate sensitivity analyses. Formal analysis, for example using logistic regression with ‘missingness’ as a response, may also be appropriate and aid interpretation.

The primary outcome will be compared between treatment groups using multi-variant logistic regression analysis, adjusting for participant age, gender and co-morbidity; regression coefficients will be considered to be significant if p-values are less than 0.05 (5% significance level). Estimates of treatment effects will be presented with 95% confidence intervals. Although generally we have no reason to expect that the clustering effects will be important for this study, in reality the data will be hierarchical in nature, with patients naturally clustered into groups. Therefore we propose to account for this by generalizing the conventional linear (fixed-effects) logistic regression approach to a more general mixed-effects modelling. i.e. a random effect is included in the model to account for heterogeneity. This analysis will be presented in addition to the conventional fixed effects model. All analyses will be conducted on an intention-to-treat (ITT) basis, and reported as such; additional per-protocol analyses will be undertaken and reported if these prove to be informative.

Any subsequent amendments to this initial SAP will be clearly stated and justified. Interim analyses will be performed only where directed by the data monitoring committee (DMC). The routine statistical analysis will mainly be carried out using R (http://www.r-project.org/) and S-PLUS (http://www.insightful.com/).

## Discussion

Surgical site infection in this population of patients is associated with increased mortality, morbidity, and pain, which are well documented in the international literature [20]. For patients undergoing an elective procedure, reduction of postoperative complications should be a targeted objective for all doctors [21].

A targeted intervention ideally needs to be easily adopted, low-intensity, independent of local hospital policy, and surgeon factors [22]. One of the great advantages of this trial is the ease with which the intervention could be applied pragmatically into any NHS hospital. The number of patients who develop SSI will continue to increase as the number of these procedures increases, and therefore it is important that interventions optimise outcomes after surgery are evaluated. Pragmatic RCTs represent the highest level of evidence to assess the clinical effectiveness of a new intervention. However, there are very few well-designed and sufficiently powered RCTs of orthopaedic interventions, in particular assessing SSI. Many orthopaedic trials fail to be delivered due to lack of scientific rigor in study design and execution. NASS is a pragmatic trial with an appropriate sample size calculation, double blind, with clearly defined inclusion and exclusion criteria, and information on statistical analysis considered appropriately. The trial has been specifically designed to demonstrate a clinically important difference for patients, surgeons and within the wider setting of the NHS.

The primary aim of the NASS trial is to determine if the addition of a triclosan coated suture to the standard regime within an NHS trust setting can significantly reduce SSI at 30 days after hip and knee replacement. The results from this trial will inform evidence-based recommendations for patients undergoing these common procedures in the UK and around the world. If a triclosan coated suture is found to be an effective intervention, its implementation into clinical practice could reduce SSI in a broad range of surgical specialities, and therefore improve long-term outcomes for patients undergoing a broad range of surgical procedures.

## Abbreviations

SSI: Surgical site infection; RCT: Randomised controlled trial; HPA: Health Protection Agency; TKR: Total knee replacement; NHS: ational Health Service; THR: Total hip replacement; NASS: Northumbria Arthroplasty Suture Study; CDC: Centers for Disease Control and Prevention; ONS: Office of National Statistics; DVT: Deep Vein Thrombosis; PE: Pulmonary Embolism; TIA: Transient Ischemic Attack; GIB: Gastrointestinal bleeds; UR: Urinary Retention; UTI: Urinary tract infection: MI: Myocardial Infarction; RTI: Pneumonia ARF, Acute Renal Failure; MCAR: Missing completely at random (MCAR); ITT: Intention-to-treat; DMC: Data monitoring committee.

## Competing interests

The authors declare that they have no competing interests.

## Authors’ contributions

All authors conceived and designed the study. All authors drafted the manuscript, revised it critically for important intellectual content and have given final approval of the version to be published. All authors read and approved the final manuscript.

## Pre-publication history

The pre-publication history for this paper can be accessed here:

http://www.biomedcentral.com/1471-2474/15/237/prepub
